# Brain Weight and Intelligence

**Published:** 1889-01

**Authors:** 


### BRAIN WEIGHT AND INTELLIGENCE.

He does science a favor who dispels the least of still existing scientific superstitions. A scientific superstition has long prevailed in regard to the weight of the brain as compared with the weight of the body in man and other animals. Most physiologists hold that the weight of the human brain is one-fortieth of that of the body, and that such a proportion is not attained by any other animal. This flattering superstition is dispelled by some recent investigations of Dr. Joseph L. Handcock (*American Naturalist*, XXII. p. 537, June, 1888) on the weight of the body and brain of birds. He finds that in the smaller and more intelligent birds the weight of the brain rises to one-thirteenth that of the body in *Stetophaga ruticilla* (American redstart), and to one-seventeenth in *Regulus calendula* (ruby crowned kinglet) and *Parus atricapillus* (black capped chickadee); and in the larger and more sluggish of the tribe it often remains higher than in man or any carnivora.

